# Adenosine diphosphate released from stressed cells triggers mitochondrial transfer to achieve tissue homeostasis

**DOI:** 10.1371/journal.pbio.3002753

**Published:** 2024-08-20

**Authors:** Hao Li, Hongping Yu, Delin Liu, Peng Liao, Chuan Gao, Jian Zhou, Jialun Mei, Yao Zong, Peng Ding, Meng Yao, Bingqi Wang, Yafei Lu, Yigang Huang, Youshui Gao, Changqing Zhang, Minghao Zheng, Junjie Gao

**Affiliations:** 1 Department of Orthopaedics, Shanghai Sixth People’s Hospital Affiliated to Shanghai Jiao Tong University School of Medicine, Shanghai, China; 2 Institute of Microsurgery on Extremities, and Department of Orthopedic Surgery, Shanghai Sixth People’s Hospital Affiliated to Shanghai Jiao Tong University School of Medicine, Shanghai, China; 3 Department of Orthopedic Surgery, The First Affiliated Hospital of Xiamen University, School of Medicine, Xiamen University, Xiamen, China; 4 Centre for Orthopaedic Research, Medical School, The University of Western Australia, Nedlands, Australia; 5 Perron Institute for Neurological and Translational Science, Nedlands, Australia; University of Washington, UNITED STATES OF AMERICA

## Abstract

Cell-to-cell mitochondrial transfer has recently been shown to play a role in maintaining physiological functions of cell. We previously illustrated that mitochondrial transfer within osteocyte dendritic network regulates bone tissue homeostasis. However, the mechanism of triggering this process has not been explored. Here, we showed that stressed osteocytes in mice release adenosine diphosphate (ADP), resulting in triggering mitochondrial transfer from healthy osteocytes to restore the oxygen consumption rate (OCR) and to alleviate reactive oxygen species accumulation. Furthermore, we identified that P2Y2 and P2Y6 transduced the ADP signal to regulate osteocyte mitochondrial transfer. We showed that mitochondrial metabolism is impaired in aged osteocytes, and there were more extracellular nucleotides release into the matrix in aged cortical bone due to compromised membrane integrity. Conditioned medium from aged osteocytes triggered mitochondrial transfer between osteocytes to enhance the energy metabolism. Together, using osteocyte as an example, this study showed new insights into how extracellular ADP triggers healthy cells to rescue energy metabolism crisis in stressed cells via mitochondrial transfer in tissue homeostasis.

## Abstract 2

Cell-to-cell mitochondrial transfer within the osteocyte dendritic network regulates bone tissue homeostasis, but the underlying mechanisms remain unknown. This study show that osteocytes release ADP under stress conditions, triggering mitochondrial transfer to achieve tissue homeostasis.

## Introduction

Increasing evidence indicated recently that mitochondria undergo dynamic intercellular transfer between cells [1]. The horizontal mitochondrial transfer has been observed in vivo and proved to play roles in physiological [2,3] and pathophysiological conditions [4]. Their impact in physiological and pathological conditions has been implied in various tissues and organs [5]. Therapeutic strategies for restoration of mitochondrial functions have been developed. One of the emerging trends is mitochondrial transfer and transplantation, which have become a promising therapeutic option for treatment of diseases such as ischemic diseases [6,7], reperfusion injuries [8], and neural disorders [9]. Our previous study demonstrated that osteocytes sustain their viability by transferring healthy mitochondria to neighboring stressed osteocytes [10]. While several studies have shown the role of intercellular mitochondria movement toward cells with dysfunctional mitochondria in tissue homeostasis [11–13], it is not clear what triggers the transfer of mitochondria in cells.

During aging, mitochondrial membrane undergoes profound architectural changes, including inner membrane vesiculation and ATP synthase dimer dissociation, which in turn impairs adenosine diphosphate (ADP) turnover in cells. Subsequent reactive oxygen species (ROS) accumulation initiates various types of cellular damage [14,15]. Mitochondrial dysfunction plays a vital role in various physiological and pathological processes, including age-related degeneration. Abnormalities in mitochondrial structures, components, and functions precipitate diseases of nervous [16], cardiovascular [17], digestive [18], and immune system [19]. In this study, we demonstrated a mechanism of triggering mitochondrial transfer between cells. It is also shown that mitochondrial transfer play roles in maintaining bone homeostasis. Extracellular ADP acts as a chemical signal to trigger intercellular mitochondrial transfer between osteocytes, interacting with the nucleotide receptors, *P2Y2* and *P2Y6*, on neighboring cells in a paracrine manner. Mitochondrial transfer is initiated to rescue energy metabolism crisis in stressed cells. Our results revealed a novel mechanism of cell–cell interaction in maintaining tissue homeostasis through mitochondrial transfer.

## Results

### Aging-associated mitochondrial impairment leads to extracellular ADP release

In senescent cells, the structure of mitochondria changes profoundly, and the functions of mitochondria in providing ATP decrease gradually during the process of aging [14]. Notably, in aged tissue, cells with injured mitochondria tend to release various damage-related factors including nucleotides [20], which leads to an elevation of concentration of extracellular nucleotides [21]. Osteocytes accumulate molecular damage over time during their long life span. To examine the molecular profile changes of aging osteocytes, we conducted transcriptomic analysis on cortical bone particles from 1-month (young) or 18-month (aged)-old mice. Gene ontology (GO) enrichment analysis of significantly down-regulated genes (the genes selected were down-regulated > 2-fold with *P* < 0.05) (S1 Fig) suggested impeded cell metabolism in aging osteocytes, which was indicated by dysregulation of pathways associated with energy storage, energy derivation, and oxidative stress responses, as well as down-regulation of transcripts linked with ATP binding, mitochondrial outer membrane, and NAD^+^ ADP-ribosyltransferase activity (S1 Fig).

**Fig 1:**
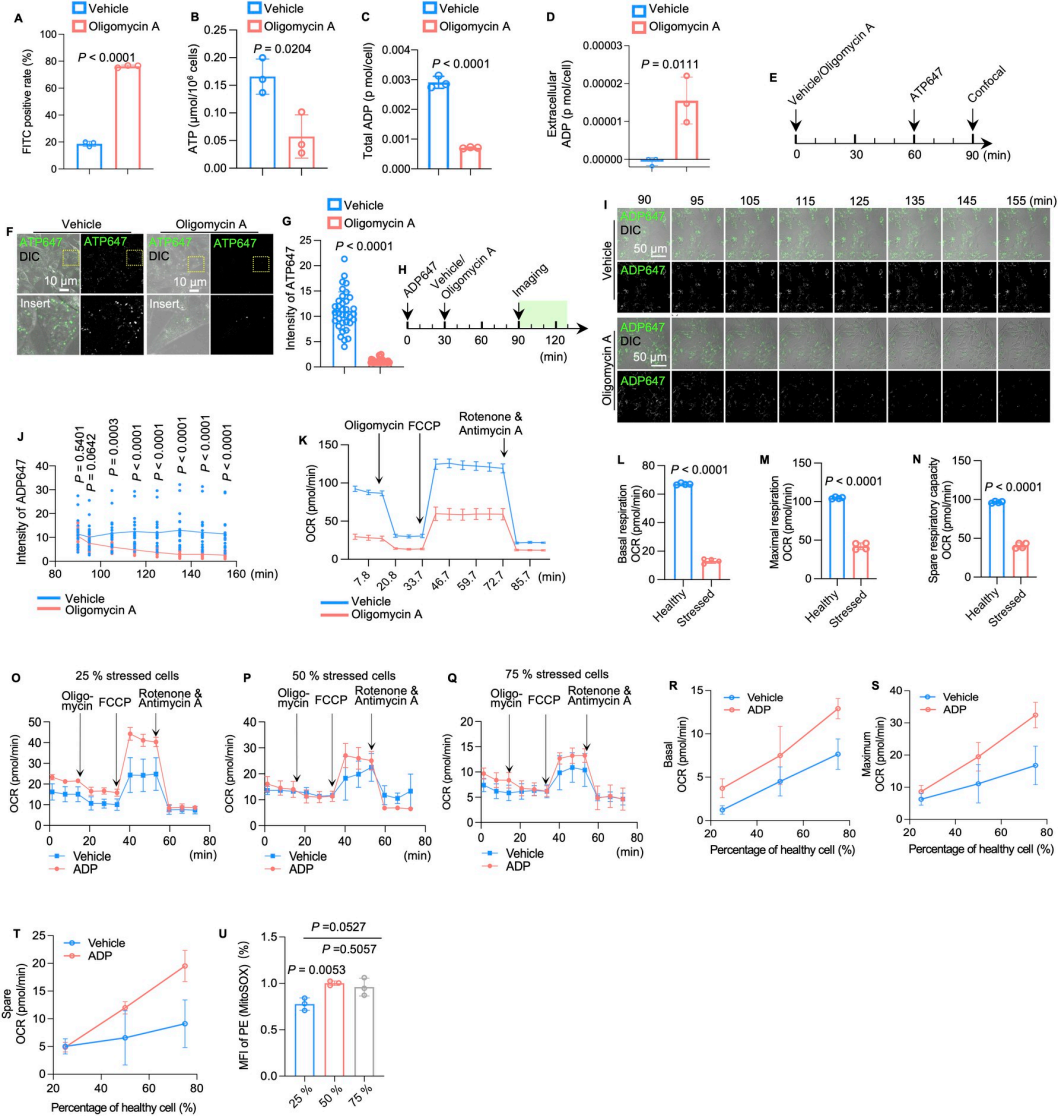
Impaired mitochondria contribute to ADP release and ADP restores the respiratory capacity of stressed cells co-cultured with healthy cells. (A) ROS (FITC)-positive rate of MLO-Y4 cells treated with vehicle or oligomycin A in flow cytometry analysis (*n* = 3). (B) The level of endogenous ATP in MLO-Y4 cells treated with vehicle or oligomycin A (*n* = 3). (C) Total ADP levels in cell lysates from MLO-Y4 cells treated with vehicle or oligomycin A (*n* = 3). (D) The concentration of extracellular ADP in supernatants from MLO-Y4 cells treated with vehicle or oligomycin A (*n* = 3). (E) Diagram of the incubation of ATP647 in in MLO-Y4 cells treated with vehicle or oligomycin A. (F) Confocal live cell imaging of ATP 647 in MLO-Y4 cells treated with vehicle or oligomycin A. (G) Quantitation of ATP 647 fluorescence intensities in MLO-Y4 cells treated with vehicle and oligomycin A (*n* = 35). (H and I) Schematic and representative time course confocal imaging with 10-min intervals of ADP647-incubated MLO-Y4 cells, with or without oligomycin A treatment. (J) Time course analysis of fluorescence intensity of ADP647 in cells treated with vehicle or oligomycin A (vehicle *n* = 21, oligomycin A *n* = 20). (K–N) OCR analysis of MLO-Y4 cells treated with vehicle or oligomycin A (*n* = 4). (O–Q) OCR of the co-culture model containing 25%, 50%, or 75% stressed MLO-Y4 cells. Each model was treated with vehicle or ADP (*n* = 3). (R–T) The effect of ADP on basal, maximal, and spare respiratory capacity of models containing different ratios of stressed cells. (U) Flow cytometry analysis showing the percentage of MitoSOX-positive cells in the co-culture model of different amounts of stressed cells after adding ADP compared to the percentage of MitoSOX-positive cells in the co-culture model of different amounts of stressed cells after adding the vehicle; (*n* = 3) min stands for minute. The data underlying the graphs shown in the figure can be found in S1 Data. ADP, adenosine diphosphate; OCR, oxygen consumption rate; ROS, reactive oxygen species.

To establish a model of osteocytes with dysfunctional mitochondria in vitro, we specifically inhibited ATP synthase by administering oligomycin A [22–24] in an osteocyte-like cell line (MLO-Y4 cells). Accumulation of ROS (Fig 1A) together with decreased endogenous ATP levels (Fig 1B) indicated impaired function of mitochondria in oligomycin A-treated MLO-Y4 cells [25,26]. In mitochondria with impaired aerobic respiration, ATP tends to be dephosphorylated to ADP and subsequently released out of mitochondria [27]. Conversely, we showed that osteocytes treated with oligomycin A release ADP into the supernatant, while the total ADP levels were decreased in the stressed cells (Fig 1C and 1D). To evaluate ATP turnover in osteocytes with dysfunctional mitochondria, we then employed a fluorescence ATP analog (Alexa Fluor 647 ATP, ATP647), which can be internalized by cells to monitor ATP turnover [28–30]. After incubation with ATP647 (Fig 1E), confocal live cell imaging revealed a remarkable decrease in fluorescence intensity in oligomycin A-treated MLO-Y4 cells compared with the vehicle group cells (Fig 1F and 1G). Next, we examined the colocalization between ATP647 and ATP synthases. We immunostained MLO-Y4 cells with an antibody against ATP5β [31], which localizes to the mitochondrial membrane and exists as a subunit of the Fo complex to regulate the production of ATP [32,33]. In native osteocytes, nearly 25% of ATP647 signals colocalized with ATP5β. However, the rate of colocalization decreased to 8% in oligomycin A-treated osteocytes (S2A and S2B Fig). To explore the release of ADP, we first incubated osteocytes with a fluorescent ADP analog (EDA-ADP-ATTO-647 N, ADP647) and replaced the medium with medium containing oligomycin A to impair mitochondrial function (Fig 1H). Live cell imaging with 10 min intervals revealed a decrease in fluorescence intensities in oligomycin A-treated MLO-Y4 cells compared to the vehicle group cells (Fig 1I and 1J), indicating that dysfunctional mitochondria in MLO-Y4 cells induce extracellular ADP release.

### Extracellular ADP mediates healthy cells rescuing stressed cells through restoration of the respiratory chain capacity

Next, we explored the role of released nucleotides in maintaining the homeostasis of the osteocyte network. We adopted an MLO-Y4 cells co-culture system for the mitochondrial stress test using different ratios of healthy and stressed cells. The mitochondrial stress test showed that oligomycin treatment decreased mitochondrial activity, as the oxygen consumption rate (OCR) dropped drastically in these stressed cells (Fig 1K–1N). When the number of healthy MLO-Y4 cells decreased, the OCR dropped dramatically, indicating that there were fewer healthy cells to maintain homeostasis (Fig 1O–1T). Moreover, ADP supplementation enhances mitochondrial respiratory activity. However, when there were less than 50% healthy cells within the system, the effect of ADP treatment was less obvious (Fig 1O–1T). Next, we examine whether ADP administration could alleviate ROS levels in the same co-culture system by conducting cytometry quantification. We showed that after adding ADP, the ROS level (MitoSOX) decreased compared to that of the same system without ADP treatment. Similarly, when there were less than 50% healthy cells, this effect was not obvious (Fig 1U). Moreover, the effect of ADP in alleviating ROS could not be observed in stressed cells (S3 Fig). These results suggested that the effect of ADP restoring the respiratory chain capacity requires the presence of a sufficient number of healthy cells.

### Extracellular ADP induces mitochondrial transfer from healthy cells to stressed cells by binding to nucleotide receptors P2Y2/6

**Fig 2:**
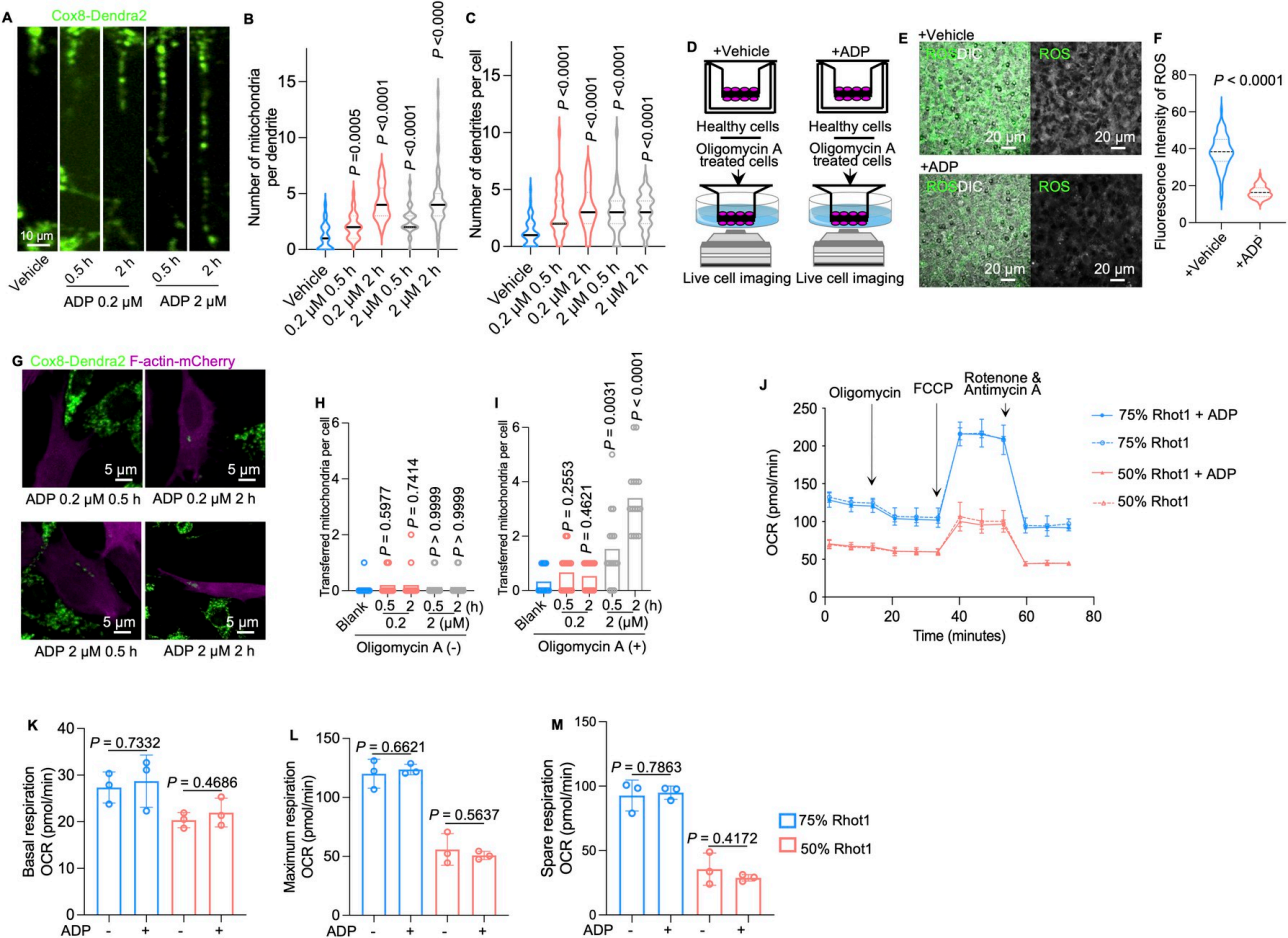
ADP activates mitochondrial transfer and alleviates ROS levels in stressed cells. (A) Representative confocal live cell images of Cox8-labeled (Dendra2) mitochondria in MLO-Y4 cell dendrites treated with vehicle, 0.2 μm or 2 μm ADP with 0.5 h or 2 h incubation. (B) Number of mitochondria per dendrites in MLO-Y4 cells. (C) Number of dendrites per cell. (D) Diagram of the Transwell 3D co-culture model (inner side: healthy osteocytes; outer side: stressed cells). (E and F) Live cell imaging and semiquantitative analysis showed attenuated ROS accumulation in the 0.2 μm ADP treatment group (*n* = 75). (G) Representative confocal images of co-culture systems of stressed cells (F-actin labeled: mCherry) and healthy cells (Cox8 labeled: Dendra2), which are stimulated by ADP. (H) Number of transferred mitochondria per unstressed cell with or without stimulation of ADP (*n* = 15). (I) Number of transferred mitochondria per stressed cell, with or without stimulation of ADP (*n* = 15). (J) OCR of the coculture model containing 75%, 50% healthy cells with Rhot1 being knocked down using shRNA (*n* = 3). (K–M) Basal, maximal, and spare respiratory capacity of models containing different ratios of healthy cells with or without ADP stimulation. Oligo stands for oligomycin A. OCR stands for oxygen consumption rate. Rhot1 stands for Rhot1-knocked-down cells. FCCP stands for mitochondrial oxidative phosphorylation uncoupler; Carbonyl cyanide 4-(trifluoromethoxy) phenylhydrazone; h stands for hour. The data underlying the graphs shown in the figure can be found in S2 Data. ADP, adenosine diphosphate; ROS, reactive oxygen species.

Our previous study demonstrated that through mitochondrial transfer, stressed/aged osteocytes can obtain mitochondria from healthy/young osteocytes [10]. Therefore, we investigated whether released ADP could trigger mitochondrial transfer in neighboring osteocytes. Previously, studies have shown that cells release nucleotides at a wide range of concentrations from 100 nM to more than 1 μm, and stressed cells tend to release higher concentrations [34,35]. We thus used both low (0.2 μm) and high (2 μm) concentrations of ADP to test the effect of extracellular ADP on osteocytes. We used MitoTracker dye and a cell line with stable expressed Dendra2 fluorescent mitochondria to monitor mitochondrial transfer. Confocal live cell imaging of osteocyte dendrites (Figs 2A, S4A, and S5) demonstrated that ADP at both low and high concentrations enhances the number, size, and confluence of dendritic mitochondria as well as the length of dendrite and the number of dendrites in MLO-Y4 cells, as compared with those in the vehicle-treated MLO-Y4 cells (Figs 2B, 2C, and S4B–S4G). In the Transwell co-culture model, we seeded vehicle- or oligomycin A-treated osteocytes on each side of the Transwell membrane (upper side: healthy cells; lower side: stressed cells) whose pores were large enough (8 μm) to allow the dendrites to going through instead of the entire cell body. Then, we stimulated healthy osteocytes on upper side with vehicle or ADP (Fig 2D). Remarkably, compared to the stressed osteocytes in the vehicle group, the stressed osteocytes in the ADP-treated group showed recovery of mitochondrial metabolic function, as indicated by the decreased level of ROS (Fig 2E and 2F). Furthermore, ADP treatment in oligomycin A-treated cells could not alter the ROS level (S6A and S6B Fig). Next, we validated whether mitochondrial transfer occurs between healthy and stressed cells, using a co-culture system of MLO-Y4 cell line labeled F-actin with mCherry and MLO-Y4 cells labeled with Cox8. F-actin labeled MLO-Y4 cells was treated with or without oligomycin A, acting as the recipient cells of mitochondria. After being stimulated by 0.2 μm or 2 μm ADP in 0.5 h or 2 h, confocal imaging assessment demonstrated that more exogeneous mitochondria (green) in recipient cells (magenta) when the recipient cells were stressed by oligomycin A (Fig 2G–2I). To confirm the relocation of Cox8-labeled mitochondria in F-actin-labeled MLO-Y4 cells is due to mitochondrial transfer between cells, we constructed another cell line by knocking down Rhot1, a vital protein responsible for mitochondrial movement (S7 Fig). We used different ratio of cell mix at 75% Rhot 1 KO cells to 25% of stressed cells and 50% to 50% of Rhot 1 KO cells to 25% of stressed cells in co-culture as in Fig 1O and 1P. The results of the co-culture seahorse study showed that stressed cells fail to recover the respiratory capacity, suggesting that when mitochondria transfer was blocked, ADP stimulation cannot restore the respiratory capacity of the co-culture system (Fig 2J–2M). These results indicated that released ADP from stressed cells signals mitochondrial transfer from neighboring healthy osteocytes.

**Fig 3:**
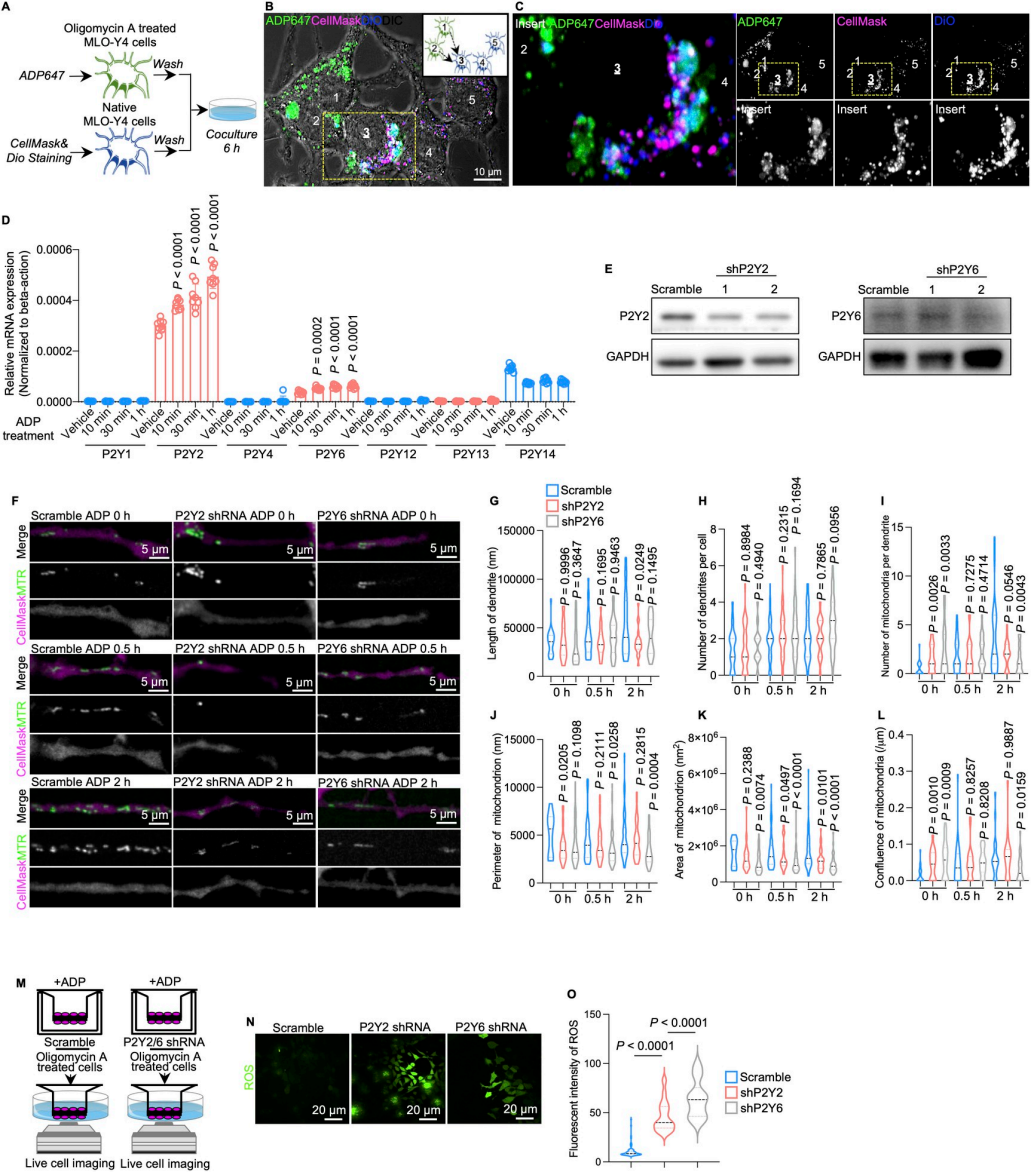
P2Y receptors transduce ADP signals in healthy mitochondria donor cells. (A) Diagram of the 2D osteocyte co-culture system demonstrating ADP release and binding. (B) After 6 h of co-culture, a representative confocal image (left) shows the fluorescence of ADP647, CellMask and DiO. Diagram (right) demonstrates the direction (arrows) of release and binding of ADP647 from stressed cells (1 and 2) to the dual-color labeled osteocytes (3, 4, and 5). (C) Insert confocal images show colocalization of ADP647 and CellMask/DiO on the membrane of healthy cells [3]. (D) Relative mRNA expression of P2Y receptors in MLO-Y4 cells after treatment with additional ADP (*n* = 8). Parameters in group of 10 min, 30 min, and 1 h were compared to those in group of vehicle, according to which *P* values have been calculated. (E) Immunoblots of P2Y2 (top) and P2Y6 (bottom) in MLO-Y4 cells after the 2 receptors were knocked down by shRNA lentivirus (*n* = 3). (F) Representative confocal live cell images of MTR-labeled MLO-Y4 cells (scramble and *P2Y2/P2Y6* knockdown) dendrites treated with vehicle and 2 μm ADP. Images of 0-h, 0.5-h, and 2-h ADP treatment were collected. (G–L) Quantification of mitochondrial parameters (number of mitochondria per dendrite, length of mitochondria, average length of mitochondria) in P2Y-shRNA MLO-Y4 dendrites treated with vehicle and 2 μm ADP. Parameters in group of shP2Y2 and shP2Y6 were compared to those in group of Scramble, according to which *P* values have been calculated. (M) Diagram of the Transwell 3D co-culture model (inner side: MLO-Y4 cells scramble/P2Y-shRNA; outer side: stressed cells). (N) Representative confocal images of ROS fluorescence intensity in stressed cells on the outer side of the 3D co-culture model in (M). (O) Quantification of the fluorescence intensity of ROS in MLO-Y4 cells treated with oligomycin A on the outer side. The data underlying the graphs shown in the figure can be found in S3 Data. ADP, adenosine diphosphate; ROS, reactive oxygen species.

To investigate the receptor of extracellular ADP on neighboring osteocytes, we employed a 2D co-culture system. First, we dual-color labeled the plasma membrane of recipient osteocytes with DiO (blue) and CellMask (red), and then co-cultured them with oligomycin A stimulated osteocytes containing ADP647 (Fig 3A). Intriguingly, after 6 h of co-culture, confocal live cell imaging detected the fluorescence intensities of ADP647 on the dual-color labeled osteocyte membranes [3], indicating that ADP released from stressed osteocytes (1 and 2) could bind to the membrane of neighboring healthy osteocytes (Fig 3B and 3C). These results suggest that extracellular ADP released from stressed osteocytes into the extracellular matrix could potentially interact with to neighboring cell through membrane contact.

**Fig 4:**
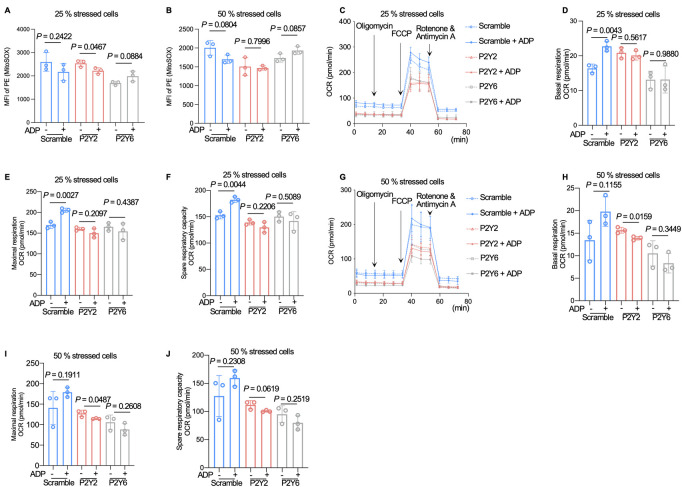
*P2Y2/P2Y6* knockdown blocks the effect of ADP on stressed cells and signal transduction. (A and B) Flow cytometry analysis showing the mean fluorescence intensity of MitoSOX-positive cells in co-culture systems containing 25% or 50% stressed cells with or without the stimulation of additional ADP (*n* = 3). (C–F) OCR of the co-culture system in (A) (25% stressed cells). Quantified basal (D), maximal (E), and spare (F) respiratory capacity were evaluated with or without additional ADP treatment (*n* = 3). (G–J) OCR of the co-culture system in (B) (50% stressed cells). Quantified basal (H), maximal (I), and spare (J) respiratory capacity were evaluated with or without additional ADP treatment (*n* = 3). The data underlying the graphs shown in the figure can be found in S4 Data. ADP, adenosine diphosphate; OCR, oxygen consumption rate.

Nucleotide-sensitive P2Y receptors, expressed in bone tissue, participate in numerous biological processes [36]. To explore how extracellular ADP triggers healthy cells for recurring cells with mitochondrial damage, we speculated P2Y receptor family may be the candidates. We thus conducted qPCR analysis on ADP (2 μm)-pretreated MLO-Y4 cells. The results showed that among the P2Y receptors, only P2Y2 and P2Y6 responded to ADP stimulation (Fig 3D). After knocking down the 2 receptors by shRNA lentivirus infection (Fig 3E), the effect of ADP stimulation on osteocyte mitochondrial transfer was less evident and even inhibited (Figs 3F–3L and S8). Using the Transwell co-culture system, we cultured the scramble cells or *P2Y2/P2Y6* knockdown cells on the upper side of the membrane and applied the ADP treatment in the upper chamber. The ROS level alone did not change in the mitochondria recipient cells on the lower side, as the knockdown cells blocked the nucleotide signals (Fig 3M–3O). In addition, we used different ratio of cell mix at 75% *P2Y2/P2Y6* knockdown cells to 25% of stressed cells and 50% to 50% of *P2Y2/P2Y6* knockdown cells to 25% of stressed cells in co-culture. After the treatment of ADP, the ROS level of the co-culture system maintained unchanged. It is suggested that deficiency of P2Y2/P2Y6 blocked the effects of ADP that we previously observed (Fig 4A and 4B). Furthermore, according to the results of co-culture seahorse experiment, additional ADP supplementation could not restore the level of OCR in this co-culture system after the knockdown of *P2Y2* or *P2Y6* even though there were enough healthy osteocytes (75% and 50% healthy *P2Y2/P2Y6* knockdown cells) (Figs 4C–4J and S9).

### Extracellular ADP enhances endoplasmic reticulum-mitochondria contacts

**Fig 5:**
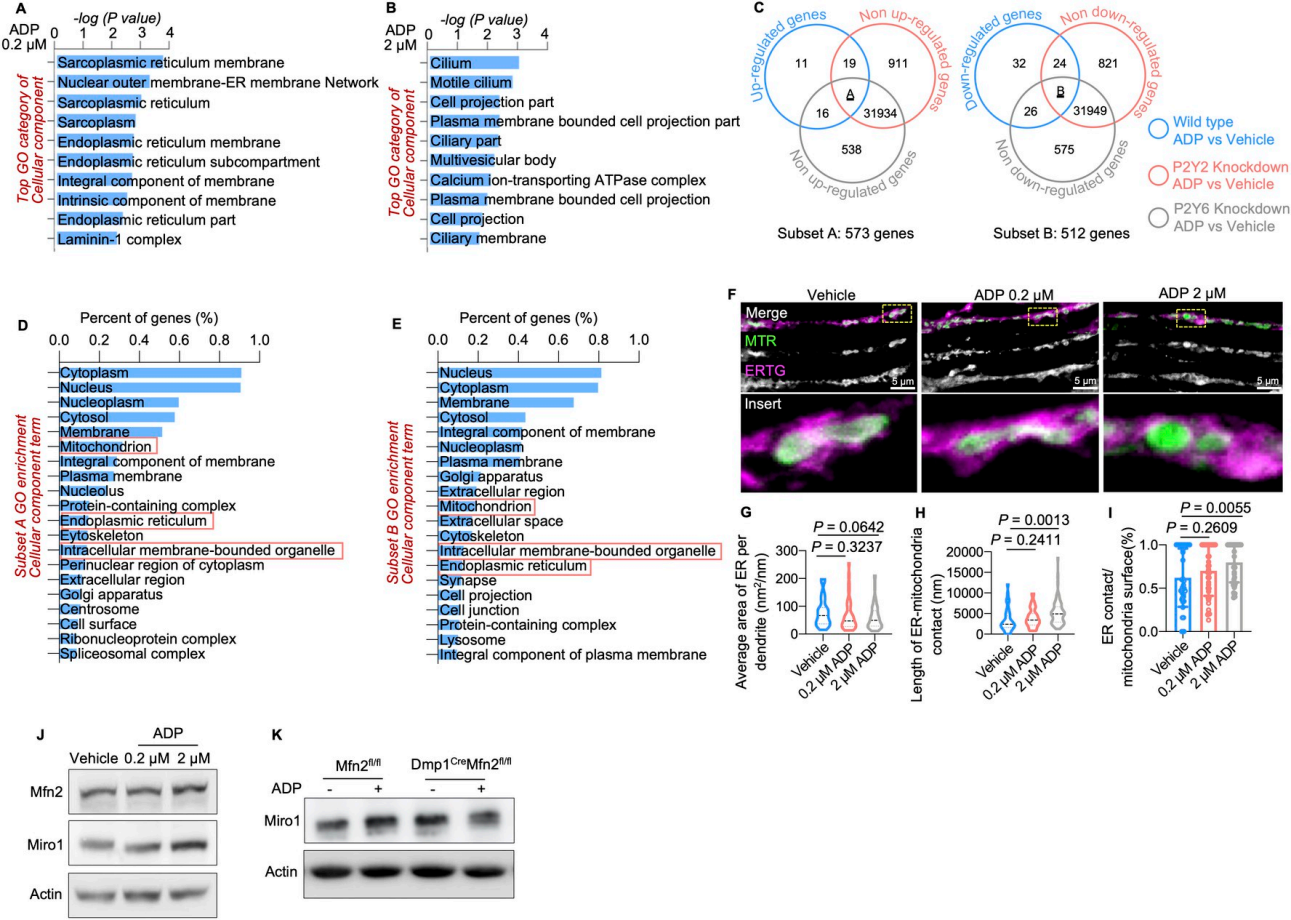
The activation of P2Y2/P2Y6 receptors promotes mitochondrial motility through Mfn2. (A and B) RNA-Seq GO analysis of significantly regulated genes showed the top regulated genes in MLO-Y4 cells treated with 0.2 μm and 2 μm ADP. (C) Venn diagram of the selection of gene subsets that might participate in the promotion of mitochondrial transfer through the membrane nucleotide receptors P2Y2 and P2Y6. Subset A: The upper left circle represents genes that are up-regulated in wild-type MLO-Y4 cells after treatment with additional ADP; the upper right and lower circles represent genes that are not up-regulated in *P2Y2/P2Y6* knockdown MLO-Y4 cells after treatment with additional ADP. Subset A is the overlap of the 3 sets of genes. Subset B: The upper left circle represents genes that are down-regulated in wild-type MLO-Y4 cells after treatment with additional ADP; the upper right and lower circles represent genes that are not down-regulated in *P2Y2/P2Y6* knockdown MLO-Y4 cells after treatment with additional ADP. Subset B is the overlap of the 3 sets of genes. (D and E) GO enrichment analysis of enriched cellular compartment terms of genes in subset A and subset B. (F) Representative images of dendrites with mitochondria (MTR) and endoplasm (ERTG) labeling in MLO-Y4 cells treated with vehicle and ADP at 0.2 μm or 2 μm. (G–I) Morphological parameter analysis based on confocal images, including the average area of ER per dendrite, length of ER-mitochondria contacts, and contact level per mitochondria. (J) Immunoblotting for Mfn2, Miro1, and Actin in MLO-Y4 cells treated with vehicle, 0.2 μm ADP and 2 μm ADP. (K) Immunoblotting for Miro1 in the osteocytes of Mfn2^fl/fl^ and Dmp1^Cre^Mfn2^fl/fl^ mice with or without ADP treatment. The data underlying the graphs shown in the figure can be found in S5 Data. ADP, adenosine diphosphate; ER, endoplasmic reticulum; GO, gene ontology.

To explore the details of mitochondrial transfer after ADP binds to P2Y2/P2Y6 receptors, an RNA-seq assay on ADP-treated MLO-Y4 cells was performed. In low concentration (0.2 μm) ADP-treated cells, GO analysis indicated that endoplasmic reticulum (ER)-associated genes were most significantly regulated (Fig 5A), and in high concentration (2 μm) ADP-treated cells, dendrite-related genes were most obviously regulated (Fig 5B). Furthermore, RNA-seq analysis of *P2Y2/P2Y6* knockdown cells was conducted to validate the effect of these 2 receptors. We combined the results of RNA-seq analysis on both wild-type and knockdown cells. Two subsets of genes were obtained by overlapping gene sets according to the results of RNA-seq analysis. Subset A and subset B contained up-regulated and down-regulated genes in WT cells after stimulation with ADP but were not up-regulated or down-regulated in knockdown cells (Fig 5C). The expression of genes in these 2 sets is not regulated by the binding of ADP to P2Y2/P2Y6 receptors. GO analysis of genes in set A and set B showed that ER- and mitochondria-associated terms were among the top enriched GO terms (Fig 5D and 5E). The results indicated that ER and osteocyte dendritic processes may be correlated with ADP-triggered mitochondrial transfer between osteocytes. Next, we investigated the status of the ER in dendrites. As the ER can directly interact with mitochondria, we stained both the ER and mitochondria by using ER Tracker Green and MitoTracker Red (MTR), respectively. Confocal live cell imaging was used to visualize the distributions of both the ER and mitochondria in MLO-Y4 cells dendrites (Fig 5F), and the levels of ER-mitochondria contact were calculated according to the length of the contact area and the perimeter of mitochondria. Although the average ER areas in dendrites were not different (Fig 5G), the length of ER-mitochondria contacts as well as the contact levels per mitochondria in dendrites were significantly increased in the high-concentration ADP-treated group (Fig 5H and 5I). Next, contact levels were further validated by the stabilized ER-mitochondria tether proteins Mfn2 and Miro1 (Fig 5J and 5K). Mfn2 mediates ER-mitochondria contacts, which have been shown to regulate mitochondrial transfer between osteocytes [18]. These results suggested that extracellular ADP triggers mitochondrial transfer by regulating ER-mitochondria contacts.

### ADP release in aging acts as a signal for mitochondrial transfer

**Fig 6:**
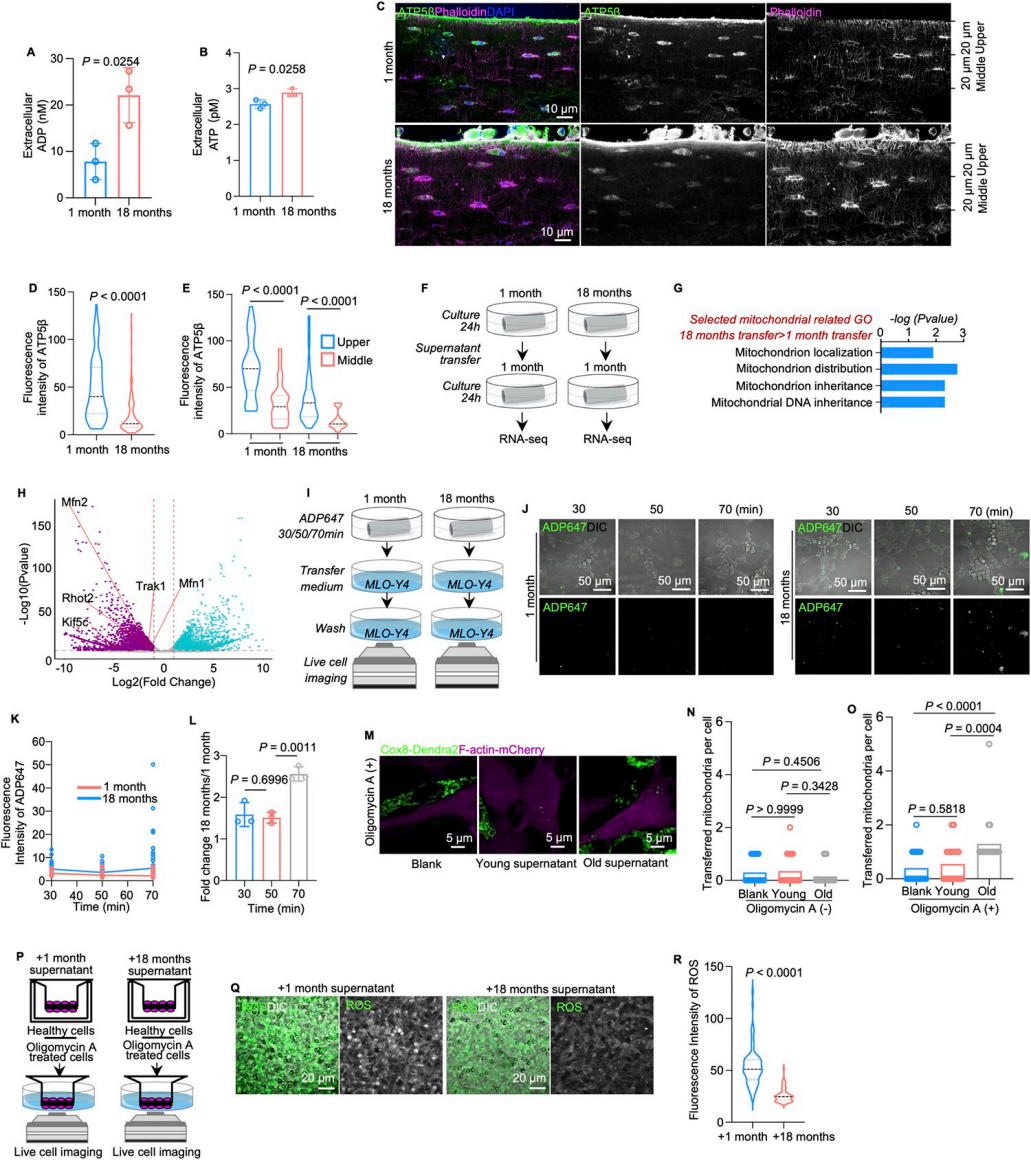
Impaired mitochondria in aged osteocytes contribute to ADP release by compromising membrane integrity. (A and B) The level of extracellular ADP and ATP in the bone fragment supernatant of 1-month and 18-month mice (*n* = 3). (C) Immunostaining of ATP5β and confocal imaging of cortical bone from 1-month-old and 18-month-old mice. The sectional area is divided into 2 parts along the longitudinal axis from the outer layer with 20 μm width for the upper and middle parts. (D) Fluorescent intensity of ATP5β in cortical bone from 1-month and 18-month mice. (E) Respective fluorescent intensity of ATP5β in upper and middle regions of cortical bone from 1-month and 18-month mice. (F) Diagram of supernatant transfer, cell culture, and RNA-Seq analysis. (G) Selected mitochondrial function-related GO analysis. Genes were significantly up-regulated in osteocytes treated with supernatant from 18-month-old mice compared with those in osteocytes treated with supernatant from 1-month-old mice. (H) Volcano map of RNA-seq expression data of genes related to mitochondrial motility in bones from 1-month-old mice treated with bone fragment supernatant from 1-month-old or 18-month-old mice. Mfn1, Mfn2, Miro2, TRAK1, and Kif5c were significantly down-regulated in samples treated with bone fragment supernatant from 1-month-old mice (*n* = 3). (I) Diagram of supernatant transfer, cell culture, and live cell imaging. Cortical bone fragments from both young and aging mice were pretreated with ADP647 and medium containing residual ADP647 that was collected following different time courses stimulated MLO-Y4 cells. (J) Representative confocal images of MLO-Y4 cells treated with medium from 1-month or 18-month mouse bone particles. (K and L) Quantification of ADP647 fluorescence intensity from 30 to 70 min in MLO-Y4 cells treated with bone fragment supernatants from 1-month-old and 18-month-old mice. (M) Representative confocal images of co-culture systems of stressed cells (F-actin labeled: mCherry) and healthy cells (Cox8 labeled: Dendra2), which are stimulated young and old osteocytes supernatant, respectively. (N) Number of transferred mitochondria per unstressed cell with stimulation of young and old osteocytes supernatants (*n* = 20). (O) Number of transferred mitochondria per stressed cell, with stimulation of young and old osteocytes supernatants. Oligo stands for oligomycin A. Young stands for osteocytes supernatant from 1-month mice. Old stands for osteocytes supernatant from 18-month mice; (*n* = 20). (P) Diagram of the 3D Transwell co-culture model. (Inner side: healthy osteocytes; outer side: stressed cells.) (Q and R) Representative live cell imaging and quantitation analysis showed attenuated ROS accumulation in the group treated with the supernatant from 18-month-old mice (*n* = 75). The data underlying the graphs shown in the figure can be found in S6 Data. ADP, adenosine diphosphate; GO, gene ontology; ROS, reactive oxygen species.

To explore whether nucleotide release increases in aged primary osteocytes, we collected supernatants from ex vivo cultured mouse bone particles and found that the supernatants from 18-month-old mice contained more nucleotides (ATP and ADP) (Fig 6A and 6B). We then examined if senescent osteocytes release nucleotides as soluble signals to regulate bone tissue homeostasis during aging.

Combining interrelated RNA-seq data sets from vehicle- or oligomycin A-treated MLO-Y4 cells with RNA-seq data sets from 1-month or 18-month primary osteocytes, we noted that nearly half (41.5%, 188 of the 453) of the significant genes regulated by oligomycin A were also regulated by the aging process in primary osteocytes (S10A Fig). These results indicate that aging osteocytes may have a similar status as oligomycin A-treated MLO-Y4 cells. This model mimicked senescent phenotypes in primary aging osteocytes according to RNA-seq analysis and western blotting, and the senescent biomarkers p16 and TNF α changed consistently (S10B Fig). Besides, compared to young bone, the expression of *P2Y2* and *P2Y6* decreased in aged bone in the analysis of RNA-seq data (S10C Fig).

Tissue immunostaining and confocal imaging analysis showed decreased ATP5β fluorescence intensity in osteocytes of aged cortical bone compared to that in osteocytes of young cortical bone (Fig 6C and 6D). Western blotting confirmed the decrease in ATP5β expression levels in the process of aging (S10D Fig). Notably, the expression of ATP5β in aging osteocytes was correlated with the distance to the periosteum, and osteocytes close to the periosteum had high ATP5β expression (Fig 6C and 6E). Due to their long lifespan, osteocytes in the same tissue can have different metabolic conditions. The results suggested that osteocytes close to the bone surface, which were newly formed, have more healthy mitochondria than osteocytes in the mid-cortex and are therefore metabolically healthier than mid-cortex osteocytes.

Next, we asked how aging osteocytes signal neighboring healthy osteocytes to initiate mitochondrial transfer. To explore this, we collected the supernatant from cultured bone fragments of 1-month-old (young supernatant) or 18-month-old mice (aging supernatant) and transferred the supernatants to cultured bone fragments (from 1-month-old mice), for subsequent RNA-seq analysis (Fig 6F and 6G). Interestingly, several genes related to mitochondrial inheritance and mitochondrial distribution were up-regulated in the bone fragment supernatant treated with aging supernatant (Fig 6H), indicating that aging supernatants enhanced mitochondrial motility. Next, we incubated ex vivo bone fragments of young (1-month-old) or aging mice (18-month-old) with ADP647. After 30, 50, or 70 min of incubation, supernatant from young and aging groups was collected and was subsequently transferred to MLO-Y4 cells (Fig 6I). In this case, more residual ADP647 in the supernatant represents less ADP maintained within the cell by primary osteocytes and vice versa. MLO-Y4 cells uptakes and turnovers the ADP647 in the supernatant (Fig 6J). At each time point, live cell imaging detected higher fluorescence intensities of ADP647 in MLO-Y4 cells cultured with supernatant from aging mice than those in MLO-Y4 cells cultured with supernatant from young mice (Fig 6J). In detail, gradually decreased intensities of ADP647 were detected in both groups from 30 to 50 min but increased from 50 to 70 min in the aging supernatant group (Fig 6K and 6L), indicating that aging primary osteocytes released more ADP that could then enter neighboring osteocytes.

Next, we stimulated MLO-Y4 cells with supernatants from young or aging mouse bone fragment ex vivo cultures (S11A Fig). Bright-field and confocal live cell imaging demonstrated more and longer dendritic mitochondria in aging supernatant-treated MLO-Y4 cells than in the young supernatant-treated MLO-Y4 cells (S11B–S11D Fig), indicating that aging enhanced mitochondrial transfer in osteocytes. Furthermore, we used the 2D co-culture system shown in Fig 2G by quantification of the exogenous mitochondria (Cox8-Dendra2) in recipient cells (F-actin-mCherry) with young or old cell supernatants. Confocal imaging assessment showed that there were more mitochondrial transfer from donor cells to the stressed cells when the co-culture system was treated with supernatants from old mice cortical bone (Fig 6M–6O). Similarly, when the recipient cells were not stressed by oligomycin A, promotion of mitochondrial transfer was not obvious after being stimulated by old supernatants. Next, by using the 3D co-culture system in Fig 3M, we co-cultured healthy MLO-Y4 cells as mitochondria donor cells with oligomycin A-treated MLO-Y4 cells as mitochondrial recipient cells and stimulated mitochondria donor cells with young (group I) or aging (group II) supernatants (Fig 6P). Semiquantitative analysis based on confocal live cell imaging demonstrated decreased fluorescence intensities of ROS in mitochondria recipient osteocytes in group II (Fig 6Q and 6R). Although the levels of ROS were not different in young or aging supernatant-treated MLO-Y4 cells (S12A and S12B Fig), supernatants from aging mice could enhance mitochondrial transfer in osteocytes to recover cell metabolism in stressed cells.

### Aging compromised cell membrane integrity leads to ADP release

**Fig 7:**
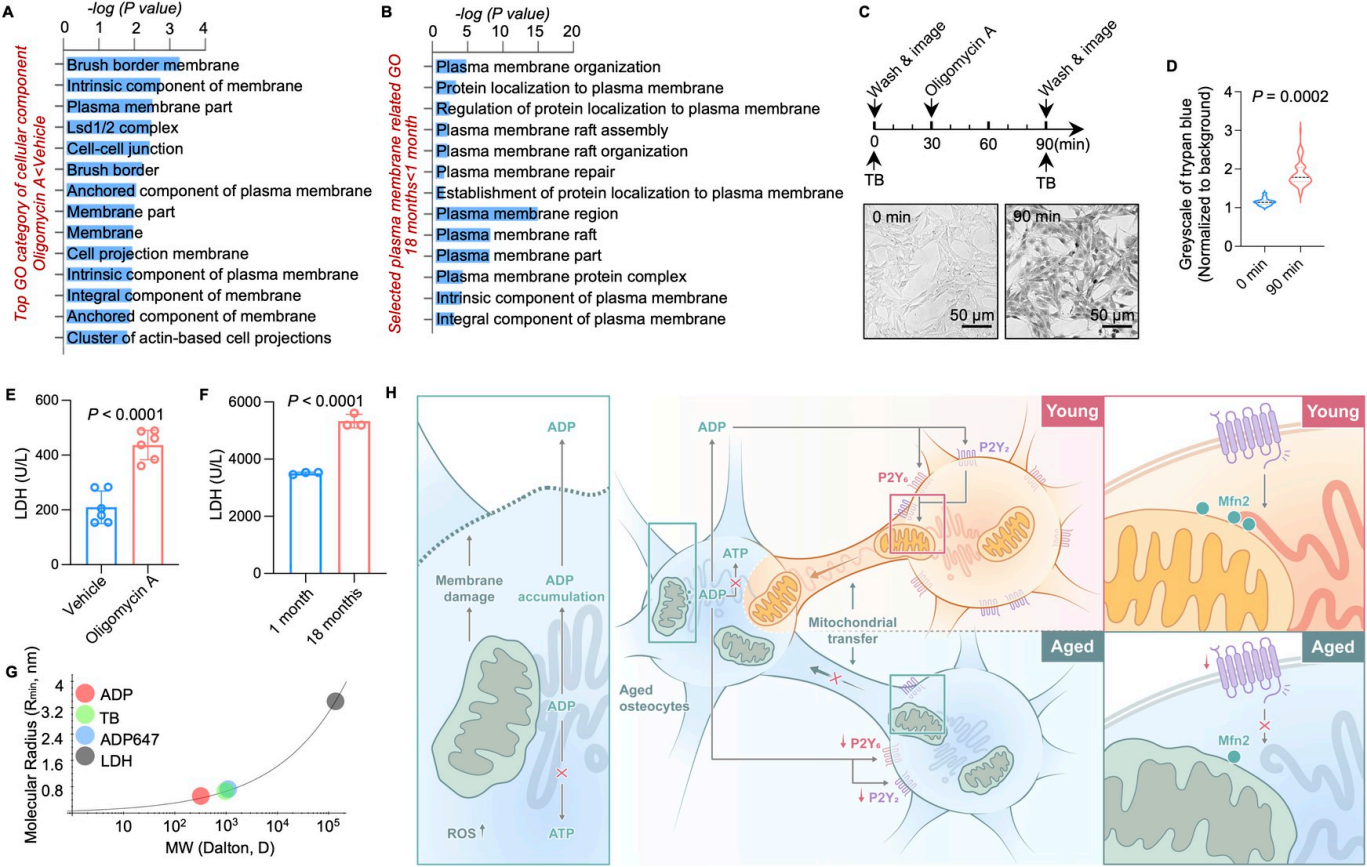
Schematic diagram of ADP as an activation signal in the dendrite networks of osteocytes during aging. (A) GO analysis of significantly regulated genes in RNA-seq showed the top regulated genes in groups treated with vehicle or oligomycin A. (B) GO analysis of significantly regulated genes in RNA-seq showed the top regulated genes in 1-month and 18-month mouse bone particles. (C) Diagram of the TB uptake assay and representative bright-field images obtained before and after oligomycin A stimulation. (D) Quantification of the normalized grayscale of TB (*n* = 80). (E) LDH leakage assay of MLO-Y4 cells treated with vehicle or oligomycin (*n* = 6). (F) LDH leakage assay of supernatant from 1-month-old or 18-month-old mouse bone particle ex vivo cultures (*n* = 3). (G) The molecular radii of LDH, TB, nucleotides, and their analogs were estimated based on their molecular weights. (H) Mitochondria of aged osteocytes are stressed during aging. Dysfunctional mitochondria cause the accumulation of ROS and ADP, which is caused by impaired synthesis of ATP. Stressed osteocytes release ADP via a disintegrated membrane to trigger neighboring healthy cells to donate their mitochondria, which are transferred to stressed osteocytes through dendrites. The nucleotide receptors P2Y2 and P2Y6 on healthy osteocytes transduce the activating ADP signal to Mfn2, which regulates mitochondrial motility. The activation of P2Y2/P2Y6 by ADP increases the mitochondria-endoplasm reticulum contacts and drives the transfer of healthy mitochondria to stressed cells. The expression of *P2Y2*, *P2Y6*, and *Mfn2* is down-regulated in aged osteocytes. Additionally, as more osteocytes in bone tissue age, the population of mitochondria donors shrinks. Overall, this process is potentially the mechanism by which bone tissue fails to maintain homeostasis when most osteocytes become senescent. This image was created by H.L. and cartonized by Ms. Lina Cao. The data underlying the graphs shown in the figure can be found in S7 Data. ADP, adenosine diphosphate; GO, gene ontology; LDH, lactate dehydrogenase; TB, trypan blue.

Next, we employed RNA-seq to explore the relationship between mitochondrial dysfunction and ADP release. GO analysis of down-regulated genes indicated that the structure and function of the plasma membrane may be compromised in oligomycin A-treated MLO-Y4 cells and aged primary osteocytes (Fig 7A and 7B), indicating a potential link between mitochondrial function and plasma membrane integrity in osteocytes. A previous study demonstrated that compromised integrity of the cell membrane contributed to the release of ATP in osteoblasts [36]. Next, we hypothesized that the release of ADP is related to the membrane integrity of osteocytes. To validate this hypothesis, a low-concentration trypan blue (TB) uptake assay was employed to examine membrane integrity [37] (Fig 7C). Bright-field microscopy showed that most MLO-Y4 cells became TB permeable following oligomycin A administration (Fig 7D), indicating impaired membrane integrity in osteocytes with dysfunctional mitochondria, which may subsequently contribute to ADP secretion. Consistently, more lactate dehydrogenase (LDH) release was measured in the culture medium of oligomycin A-treated MLO-Y4 cells and aged osteocytes (Fig 7E and 7F). Based on molecular radius estimations, the radius of the membrane lesion was large enough to allow the secretion of nucleotides as well as their analogs (Fig 7G).

## Discussion

Mitochondria is essential for cellular activates for maintaining the homeostasis in tissue functions. Mitochondria transfer between the cellular networks has recently been shown as an important mechanism of various biological and pathological process including tissue homeostasis and aging [38–42]. In our study, we identified that ADP released by stressed cells is a signal of facilitating intercellular mitochondrial transfer. Using oligomycin A-treated MLO-Y4 cells and primary aging osteocytes as a model of cells with mitochondrial dysfunction, we demonstrated that osteocytes with stressed mitochondria release ADP which acts as a rescue signal to trigger mitochondrial transfer from the surrounding healthy osteocytes. ADP binds to P2Y2 and P2Y6 receptors on neighboring cells and activate ER-mediated mitochondria transfer (Fig 7H), thus rescue energy metabolism crisis of stressed cells. Notably, aging impaired the integrity of plasma membrane, thus cause the leakage of ADP into extracellular microenvironment but decrease expression of P2Y2 and P2Y6 receptors and ER-mitochondrial contracts.

Extracellular purinergic signaling plays crucial roles in various metabolic pathways and cellular processes [43], including the cell–cell interaction during virus infections [44]. It has been shown that extracellular ATP or ADP can be released from platelets and various cell types into tissue fluids or plasma in response to various stress factors, including inflammation [45], hypoxia [46,47], and glucose deprivation [48]. Extracellular nucleotides, often released by apoptotic cells, act as chemoattractant to trigger the migration of macrophages [48,49] and subsequent phagocytic clearance [34]. Previous studies showed that osteocytes can release nucleotide through hemichannels [50], P2X7 [51], and T-type VSCC [52]. However, their extra roles in bone hemostasis are unclear. A recent study demonstrated that osteoblasts released ATP following mechanical stimulation to induce calcium responses in neighboring cells [37]. Interestingly, extracellular ADP has also been shown to enhance osteoclast-mediated bone resorption [53,54]. Deleting P2Y13 in a transgenic mouse model attenuated bone turnover rate, as demonstrated by the reduced number and activity of both osteoblasts and osteoclasts, and impaired bone remodeling rates [55]. In our study, by using exogenous fluorescence ATP and ADP analogs [28–30,56–58], we identified ADP as an intercellular signal in regulation of energy metabolism of cells in tissue homeostasis.

P2Y purinergic signaling is important for various physiological and pathological process [59–63]. These receptors transduce the nucleotide signals with different affinity towards different nucleotides. Previous studies had showed that uridine nucleotides stimulate P2Y2 and P2Y6 with high affinity, while ADP binds to these 2 receptors with lower affinity [64]. In this study, we showed for the first time that P2Y2 and P2Y6 interacts with ADP and mediates osteocyte mitochondrial transfer in bone tissue. Interestingly, the expression of the *P2Y2* and *P2Y6* receptors was down-regulated during aging (S10C Fig), suggesting these 2 members of P2Y family play important role in energy metabolism during aging.

Downstream pathway of P2Y2 and P2Y6 regulated mitochondrial transfer appeared to be via ER-mitochondrial contacts. Our previous study demonstrated a reduced number of ER-mitochondria contacts and smaller distribution of mitochondria within aging osteocyte dendritic processes [10]. Consistently, the expression of mitochondrial motility-related genes, including *Mfn1* [65,66], *Mfn2* [66], *Miro2* [67], *TRAK1* [68], and *Kif5c* [69], was significantly down-regulated in aging osteocytes compared with young osteocytes (Fig 6H), suggesting that mitochondrial transfer was impaired in aging osteocytes. These results potentially illuminate the mechanism by which P2Y2 and P2Y6 regulate the ER-mitochondria contact to initiate the mitochondrial transfer. Our study found that additional ADP stimulation increases the expression of *Mfn2* and *Miro1*, the tether protein of ER-mitochondria contacts. As a result, ADP triggers mitochondrial transfer between stressed and healthy osteocytes. Our data demonstrated for the first time that ADP released by stressed cells is crucial for tissue homeostasis.

Our study also showed that mitochondrial activities in osteocytes are determined by their anatomical locations. It seems that osteocytes close to the periosteum surface contain more ATP synthase than those in the deeper layer of cortical bone. These findings are consistent with a previous study showing that osteocytes near bone surfaces contain more active mitochondria than osteocytes in the mid-cortex [70].

Maintaining membrane structure and integrity is an energy-consuming process [71,72]. We showed that during the aging process, impaired mitochondrial function reduces membrane integrity, resulting in membrane lesions that allow the leakage of molecules much bigger than nucleotides. In support of this, a previous study indicated that enhanced energy status by ATP supplementation contributed to the maintenance of membrane integrity, as well as inhibited hydrolysis-related enzyme activity and lipid peroxidation on the cell membrane [73]. Interestingly, our study also showed that even there is an increasing releasing of ADP during aging, the reduction of *P2Y2* and *P2Y6* expression in aging osteocytes reduces the events of mitochondrial transfer between cells. When numbers of aging osteocytes accumulate and they are no longer able to support surrounding osteocytes with healthy mitochondria, which leads to the accumulation of dysfunctional mitochondria within osteocyte networks [74,75].

In conclusion, we demonstrated that ADP is an intercellular signal in regulation of energy metabolism of cells in tissue homeostasis. Released ADP plays a crucial role in mitochondrial function by targeting P2Y2 and P2Y6 receptors on neighboring cells and activating ER-mediated mitochondrial transfer. Our study provides new insights on how cells rescue energy metabolism crisis in stressed cells via mitochondrial transfer during tissue homeostasis.

## Materials and methods

### Animal procedures and cell cultures

All animal experiments were approved by the Ethical Review Board of Shanghai Sixth People’s Hospital affiliated with the School of Medicine, Shanghai Jiao Tong University (protocols #2020–0355). Long bones (femur and tibia) and the parietal bone were harvested from mice (C57BL/6J, male) aged 1 and 18 months with the periosteum, epiphysis, and bone marrow removed carefully. Then, the bones were cut into fragments or not according to the subsequent experiments and were maintained in α-minimal essential medium (α-MEM, 12561–056, Gibco) with 1% penicillin/streptomycin (P/S) or were fixed with 4% paraformaldehyde (4% PFA, Sangon Biotech). Cell numbers in bone fragments were normalized according to the amount of total protein in cell lysates.

A murine osteocyte-like cell line (MLO-Y4 cells) was maintained in α-MEM supplemented with 10% fetal bovine serum (FBS, Gibco, Thermo Fisher Scientific) and 1% (P/S) (516106, Sigma-Aldrich). Oligomycin A (5 mg, S1478, Selleck) was dissolved in DMSO to make a 50 mM stock solution. Then, the stock solution (1:1,000, 50 μm as the working concentration) or the same volume of DMSO (Vehicle) was added to fresh culture medium to stimulate the MLO-Y4 cells for 1 h at 37°C. We used these oligomycin A-treated cells to simulate dysfunctional mitochondria in osteocytes.

MLO-Y4 cells were first plated on the outer side of the membrane of the Transwell (3422, Corning Incorporated Transwell), which was then inverted and placed in a 12-well plate until the cells attached to the Transwell membrane. Then, the Transwell membrane was placed in a 24-well plate for regular use. The cells on the outer side of the membrane were soaked in culture medium containing oligomycin A or DMSO (1:200) for 1 h, which simulated mitochondrial dysfunction in the cells on the outer side. These treated cells were washed with regular culture medium 3 times and were maintained under normal conditions as a mitochondrial recipient model. The MLO-Y4 cells were then plated on the inner side of the membrane as a mitochondria donor model. The donor cells were treated with 1-month or 18-month bone fragment culture medium, ADP, or vehicle for 30 min.

### Measurement of ATP and ADP

The level of ATP and ADP was determined by colorimetric assay kit (MAK190, Sigma-Aldrich for the measurement of ATP; MAK081, Sigma-Aldrich for the measurement of ADP) following the manufacturer’s instructions. Both cellular level and the concentration in medium were determined.

### RNA sequencing sample preparation and analysis

Male C57BL/6J mice were euthanized under anesthesia. Femoral and tibia cortical bone were harvested with nonosseous tissue removed, including bone marrow. Then, the cortical bones were dissected into fragments, which were subsequently washed and centrifuged 3 times with culture medium. Nucleospin RNA II kits were used for total RNA extraction. Subsequent denaturation by magnetic oligo (dT) beads was performed before RNA sample purification. After the first-strand cDNA was obtained by reverse transcription of purified mRNA and a second cDNA was synthesized, fragmented DNA samples were blunt ended and adenylated at the 3′ ends. Adaptors were ligated to construct a library. DNA sample quantification was performed by Qubit (Invitrogen), and the samples were then sequenced by an Illumina Nova-seq 6000 instrument from Genergy Bio (Shanghai) after cBot cluster generation. Then, the raw DNA sequencing data were converted into Fastq format. In each sample, the number of fragments per kilobase of transcript per million fragments mapped (FPKM) was calculated by Cuffnorm software with a log2 transformation, which was then used to calculate the number of transcripts. The differential gene transcripts between different samples were calculated by using DESeq software. The entire set of transcripts was used as the input list for KEGG pathway analysis. The differential transcripts were selected as the candidate list, and the *P* value was calculated. Significant gene candidates were categorized based on gene functions.

### Immunofluorescence

Femurs from 1-week-old mice (C57BL/6J, male) were harvested, and the periosteum, epiphysis, and bone marrow were removed. The femur was bisected along the longitudinal axis and washed using phosphate-buffered solution (PBS) 3 times (10 min each time). The femurs were fixed in 4% PFA at room temperature for 20 min. Then, the femurs were incubated with an anti-ATP 5β antibody (1:200, A21351, Invitrogen) in 0.2% BSA-PBS solution at room temperature for 3 h. Goat-anti-mouse Alexa Fluor 488-tagged secondary antibody (1:500, A11029, Thermo Fisher Scientific) was added along with rhodamine phalloidin (1:500, R415, Thermo Fisher Scientific) and incubated at room temperature for 45 min. After that, the samples were stained with Hoechst 33342 dye (1:5,000, H3570, Invitrogen) for 15 min. After being washed 3 times with PBS, the femoral diaphyses were soaked in PBS with sectional areas being appressed onto the bottom of 35 mm glass bottom dishes (P35G-0.170-14-C, MatTek Corporation) for subsequent osteocyte confocal imaging.

MLO-Y4 cells in glass bottom dishes were stimulated with oligomycin A (50 μm) for 1 h at 37°C and then washed with fresh medium 3 times. ATP/ADP 647 (Alexa Fluor 647 ATP, A22362, Invitrogen; EDA-ADP-ATTO-647 N, 83340, Jena Bioscience) was then added at a concentration of 5 μm (1:200) in regular medium for 30 min. The fluorescence intensities were measured using confocal live cell imaging. The same stimulation and staining/imaging protocol was conducted in ex vivo cultured primary osteocytes.

After ATP live cell staining, immunostaining of ATP 5β subunits in oligomycin A/vehicle-treated MLO-Y4 cells was conducted according to the same protocol used for primary osteocytes. The colocalization between ATP647 and ATP synthases was examined by confocal microscopy imaging.

### Medium transfer experiments

Long bones (femur and tibia) and parietal bones from 1-month-old and 18-month-old mice were prepared as previously described and cut into fragments. The samples were washed with α-MEM with 2% P/S. The bone fragments from young and old mice were maintained in α-MEM supplemented with 1% P/S in 6-well plates. After 24 h of ex vivo culture in a 37°C and 5% CO_2_ humidified incubator, the culture medium in each well was collected and added to different recipients, one of which was 1-month bone fragments with the original culture medium removed before medium transfer. After another 48 h of culture, the recipient bone fragments receiving culture medium from young and old mouse bone fragments were collected for subsequent RNA-seq analysis.

MLO-Y4 cells were plated in glass bottom dishes (for confocal imaging) at low confluency to make room for the elongation of dendrites. After removal of the original culture medium, MLO-Y4 cells were stimulated with the medium (for the other experiment, the bone fragments were cultured with 5 μm ADP 647 for 30, 50, or 70 min before the medium was collected from both groups) from ex vivo cultured 1-month or 18-month bone fragments for 2.5 h (30 min for the experiment to verify the increased release of ADP) at 37°C in the incubator.

### Live cell imaging

LSM710 confocal microscopy (ZEISS) was performed for live cell imaging. Cells were stained with MitoTracker Red (1:2,000, M7512, Invitrogen) in culture medium for 20 min at 37°C protected from light, followed by 2 culture medium washes. The length of the dendrites, the number of mitochondria contained in the dendrites, and the length of a single mitochondrion were recorded during imaging. A DCFH-DA ROS assay kit (D6470, Solarbio) and MitoSOX Red (M36008, Invitrogen) were used to measure the level of ROS within the cells, which was added to α-MEM without FBS (1:1,000) and incubated with cells for 20 min at 37°C. The cells were washed with α-MEM 3 times before fluorescence was detected by confocal imaging or flow cytometry analysis. MLO-Y4 cells were first stained with DiO dye (1:200) within the medium. After 20 min, CellMask Orange (1:1,000) was then added to the medium, followed by another 10 min of co-culture. These dual-color stained cells were mixed with the same number of cells stained with ADP647 (1:200) for 30 min at 37°C in the incubator. The 2 types of cell co-cultures were maintained for 6 h at 37°C. Then, live cell imaging was used to observe the colocalization of the fluorescence. MLO-Y4 cells were stained with 0.08% trypan blue dye (0.4%, 15250061, Gibco) in culture medium. The cells were observed under bright-field microscopy. After 1 h of oligomycin A stimulation, the cells were washed with fresh medium and dyed with Trypan blue again. The changes in cellular dye uptake were evaluated by microscopy. LDH leakage assay was performed by using lactate dehydrogenase kit (A020-1-2, Nanjing Jiancheng Bioengineering Institute) according to the manufacturer’s protocols. The results are expressed in U/L. Predicted molecular radii were obtained according to previous published methods [76] based on the molecular weight of different cellular contents.

### Knock-down of *P2Y2*, *P2Y6*, and *Rhot1* via shRNA virus infection

To knock down the expression of *P2Y2* and *P2Y6*, we constructed shRNA lentiviral vectors based on the shRNAi vector pGMLV (pGMLV-zsGreen-vshRNA, Genomeditech, Shanghai, China). The shRNA-targeting sequences for *P2Y2* and *P2Y6* are as follows:

*P2Y2*-shRNA1: GGGACGAACTGGGATACAAGT

*P2Y2*-shRNA2: GCTCTCTATATCTTCCTATGC

*P2Y6*-shRNA1: GCTGCCCTTCATAGCCTTACT

*P2Y6*-shRNA2: GCCTTACTGGCTTGTTATTGT

*Rhot1*-shRNA1: GCTCAACTTCTTCCAGAGAAT

*Rhot1*-shRNA2: GATATCTCAGAGTCGGAATTT

The recombinant pGMLV-zsGreen-vshRNA-*P2Y2/P2Y6/Rhot1* were identified by PCR and DNA sequencing. Lentivirus packaging was conducted in 293T cells, followed by transfection with the shRNA-TFs into A375 cells. A scramble lentiviral vector was used as a negative control. The lentiviral particles were harvested 72 h. The supernatant was passed through 0.22-μm filter, which was collected into Eppendorf tubes and kept at −80°C for long-term storage. The optimum shRNA fragment against *P2Y2*/*P2Y6* and *Rhot1* was determined by real-time PCR. The interference of this selected shRNA on *P2Y2*/*P2Y6* and *Rhot1* was verified by fluorescent array. MLO-Y4 cells were transfected with lentivirus-carrying shP2Y2/P2Y6, shRhot1, scramble control with polybrene (10 μg/ml) for 48 h and selected by puromycin (10 μg/ml).

### Construction of fluorescent cell line

By adopting genetical modification of cells, we had developed a cell line of MLO-Y4 cells with Cox8, a subunit located in mitochondrion, labeled by Dendra2 fluorescent protein (plasmid: mito-dendra2, Addgene plasmid #55796; Vector: pHAGE Lentivirus vector) [77]. We have also labeled F-actin with stable expressed mCherry in MLO-Y4 cell line (plasmid: C1-MPAct-mCherry, Addgene plasmid #155222; Vector: pHAGE Lentivirus vector) [78], which acts as the recipients of mitochondria. MLO-Y4 cells were transfected with pHAGE-mito-dendra2 and pHAGE-MPAct-mCherry with polybrene (10 μg/ml) and screened by puromycin (10 μg/ml) for stable production of mitochondria-specific Dendra2-tagged protein and F-actin–specific mCherry-tagged protein.

### Measurements of oxygen consumption rate

The OCR was measured by Seahorse Mito Stress assay (Agilent Seahorse XFe96 Analyzers, Agilent Technologies) to evaluate oxidative phosphorylation, following the manufacturer’s operating protocols. In brief, 2 × 10^5^ cells were seeded in wells of a 96-well XF Cell Culture Microplate. In co-culture model, cells were treated with oligomycin A or vehicle before being mixed and seeded in wells. The medium was exchanged to XF DMEM medium pH 7.4 (Agilent Technologies, 103575–100) with 25 mM glucose, 2 mM glutamine, and 2 mM sodium-pyruvate (P5280, Sigma-Aldrich). Cells were incubated in this medium for 1 h at 37°C without CO_2_. OCR measurements were performed followed by the sequential addition of 2 μm oligomycin, 1 μm FCCP, and 0.5 μm rotenone/antimycin (Agilent Technologies, 103015–100). All procedures were carried out at 37°C.

### Statistical analysis

Data are presented as the mean ± standard deviation (SD). For in vitro experiments, the number of biological replicates (a minimum of 3 independent experiments) is the sample size. Statistical calculations and plotting were processed by GraphPad Prism software (version 8.0.0). Dunnett’s post hoc or Kruskal–Wallis test was performed to verify normal distribution. Significant differences between 2 groups were evaluated by two-tailed Student’s *t* test, while one-way ANOVA was used to test for significant differences among multiple groups. Significant levels are presented as exact *P* value. *P* > 0.05 was considered as statistical insignificance.

## Supporting information

S1 Fig RNA-seq and selected gene ontology (GO) analysis of bone fragments from young and old mice. Selected mitochondrial function-related GO enrichment analysis from significantly up-regulated mRNA in 18-month-old mouse bone fragments, which indicates the impairment of energy metabolic homeostasis and mitochondrial activity. The data underlying the graphs shown in the figure can be found in S8 Data.
(TIFF)

S2 Fig The colocalization rate between ATP647 and ATP synthases in vehicle or oligomycin A-treated osteocytes. (A) Confocal images and fluorescence intensity analysis indicate colocalization between ATP647 and ATP5β in MLO-Y4 cells after vehicle or oligomycin A administration. (B) The quantified rate of ATP5β colocalization with ATP647 was significantly lower in MLO-Y4 cells treated with oligomycin A. The data underlying the graphs shown in the figure can be found in S9 Data.
(TIFF)

S3 Fig ADP alone is unable to rescue the mitochondrial ROS accumulation in MLO-Y4 cells induced by oligomycin A. Flow cytometry results showing the mean fluorescence intensity of MitoSOX-positive cells in MLO-Y4 cells treated with vehicle/oligomycin A and ADP (*n* = 3). The data underlying the graphs shown in the figure can be found in S10 Data.
(TIFF)

S4 Fig ADP activates mitochondrial transfer. (A) Representative confocal live cell images of MTR-labeled MLO-Y4 cells dendrites treated with vehicle, 0.2 μm or 2 μm ADP with 0.5 h or 2 h incubation. (B–G) Quantification of mitochondrial parameters (number of mitochondria per dendrite, length of mitochondria, average length of mitochondria) in MLO-Y4 dendrites with vehicle or ADP treatment. The data underlying the graphs shown in the figure can be found in S11 Data.
(TIFF)

S5 Fig Extracellular ADP induces mitochondrial transfer between cells. Representative confocal live cell images of MTR and CellMask-labeled MLO-Y4 cells which were treated with vehicle, 0.2 μm or 2 μm ADP with 0.5 h or 2 h incubation.
(TIFF)

S6 Fig ADP treatment failed to alter the ROS level in stressed cells in absence of healthy cells. (A and B) Confocal imaging and violin plots of the fluorescence intensities show increased ROS levels in oligomycin A-treated cells, while ADP treatment did not alter the ROS level in these stressed cells. The data underlying the graphs shown in the figure can be found in S12 Data.
(TIFF)

S7 Fig Knockdown of *Rhot1* in MLO-Y4 cells by shRNA. Relative expression of Rhot1 in MLO-Y4 cells infected by Rhot1-shRNA lentivirus and its scramble control (*n* = 3). The data underlying the graphs shown in the figure can be found in S13 Data.
(TIFF)

S8 Fig Knockdown of *P2Y2/P2Y6* blocked the effect of extracellular ADP inducing mitochondrial transfer. Representative confocal live cell images of MTR and CellMask-labeled MLO-Y4 cells with *P2Y2/P2Y6* being knocked down which were treated with ADP with 0.5 h or 2 h incubation.
(TIFF)

S9 Fig Knockdown of *P2Y2/P2Y6* cannot change the respiratory capacity of osteocytes. (A) Oxygen consumption rate of scramble and *P2Y2/P2Y6* knockdown MLO-Y4 cells. Quantified basal (B), maximal (C), and spare (D) respiratory capacity were evaluated. The parameters in knock-down groups are compared to those in Scramble group, according to which *P* values are calculated (*n* = 3). The data underlying the graphs shown in the figure can be found in S14 Data.
(TIFF)

S10 Fig Impaired mitochondrial activity during aging. (A) Venn diagram of significant differentially expressed genes in cortical bone of 1-month-old and 18-month-old mice and MLO-Y4 cells treated with vehicle or oligomycin A. (B) Representative immunoblots of p16 and TNF α expression in MLO-Y4 cells after stimulation with 25 μm, 50 μm, or 100 μm oligomycin A (*n* = 3). (C) RNA-seq analysis demonstrates the expression levels of *P2Y2* and *P2Y6* in 1-month and 18-month mouse osteocytes (*n* = 3). (D) Immunoblots of ATP5β expression in cortical bone from 2-month-old, 6-month-old, and 24-month-old mice (*n* = 3). The data underlying the graphs shown in the figure can be found in S15 Data.
(TIFF)

S11 Fig Bone fragment supernatant 18-month-old mice stimulated mitochondrial transfer in MLO-Y4 cells. (A) Representative confocal imaging of the dendrites of MLO-Y4 cells, which were stimulated with bone fragment supernatant from 1-month-old or 18-month-old mice, and their mitochondria were labeled with MTR. (B–D) Quantitation of the number, length, and average length of mitochondria in dendrites of MLO-Y4 cells (*n* = 15). The data underlying the graphs shown in the figure can be found in S16 Data.
(TIFF)

S12 Fig Bone fragment supernatants from young and aged mice are unable to rescue impaired mitochondria. MLO-Y4 cells were pretreated with oligomycin A and co-cultured with bone fragment supernatants from young or aged mice (1 month or 18 months). (A) Representative confocal live cell imaging. (B) Semiquantitative analysis shows no difference in ROS fluorescence intensities between the 2 groups. The data underlying the graphs shown in the figure can be found in S17 Data.
(TIFF)

S1 Raw Images All the original blot and gel images underlying Figs 3E, 5J, 5K, S10B, and S10D. (PDF)

S1 Data The data underlying the graphs shown in Fig 1. (XLSX)

S2 Data The data underlying the graphs shown in Fig 2. (XLSX)

S3 Data The data underlying the graphs shown in Fig 3. (XLSX)

S4 Data The data underlying the graphs shown in Fig 4. (XLSX)

S5 Data The data underlying the graphs shown in Fig 5. (XLSX)

S6 Data The data underlying the graphs shown in Fig 6. (XLSX)

S7 Data The data underlying the graphs shown in Fig 7. (XLSX)

S8 Data The data underlying the graphs shown in S1 Fig. (XLSX)

S9 Data The data underlying the graphs shown in S2 Fig. (XLSX)

S10 Data The data underlying the graphs shown in S3 Fig. (XLSX)

S11 Data The data underlying the graphs shown in S4 Fig. (XLSX)

S12 Data The data underlying the graphs shown in S6 Fig. (XLSX)

S13 Data The data underlying the graphs shown in S7 Fig. (XLSX)

S14 Data The data underlying the graphs shown in S9 Fig. (XLSX)

S15 Data The data underlying the graphs shown in S10 Fig. (XLSX)

S16 Data The data underlying the graphs shown in S11 Fig. (XLSX)

S17 Data The data underlying the graphs shown in S12 Fig. (XLSX)

## Abbreviations

α-MEM: α-minimal essential medium
ADP: adenosine diphosphate
ER: endoplasmic reticulum
FBS: fetal bovine serum
GO: gene ontology
LDH: lactate dehydrogenase
OCR: oxygen consumption rate
PBS: phosphate-buffered solution
PFA: paraformaldehyde
P/S: penicillin/streptomycin
ROS: reactive oxygen species
SD: standard deviation
TB: trypan blue
