# Supplementary figures and images for: Adenosine diphosphate released from stressed cells triggers mitochondrial transfer to achieve tissue homeostasis
Source: PLoS Biol. 2024 Aug 20;22(8):e3002753. doi: 10.1371/journal.pbio.3002753 (PMC11335167; doi:10.1371/journal.pbio.3002753)

Fig 3E

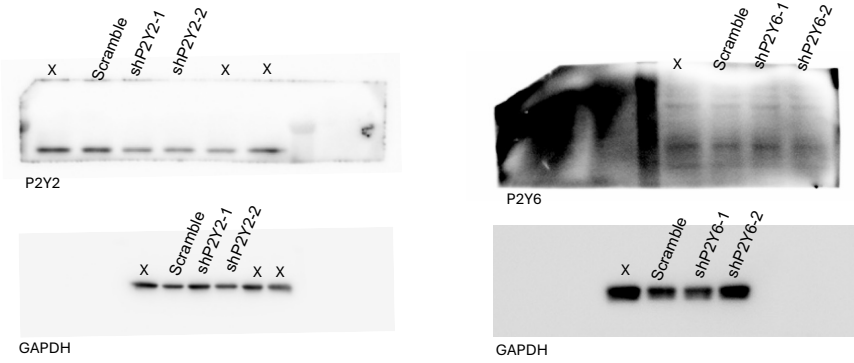

Fig 5J

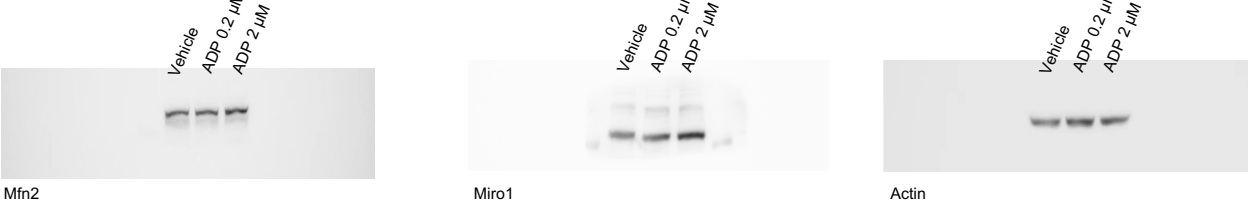

Fig 5K

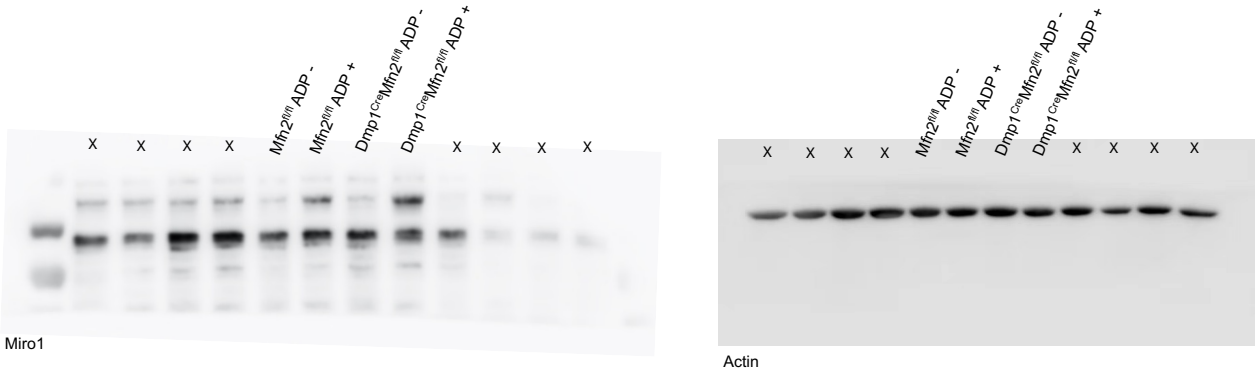

S10 B

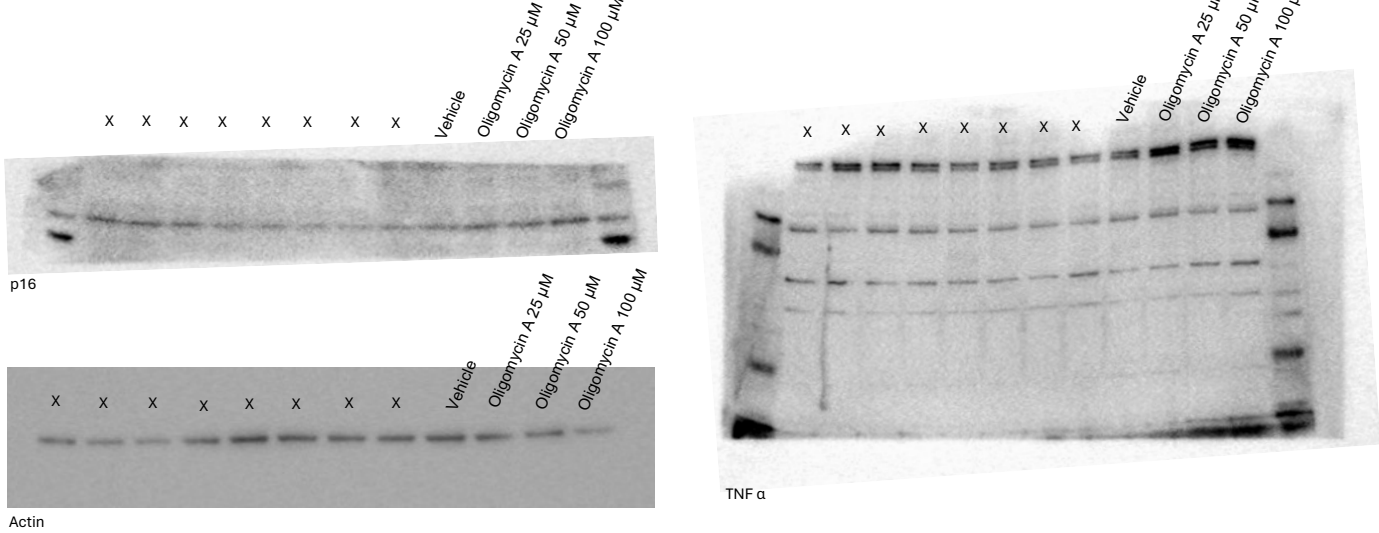

S10 D

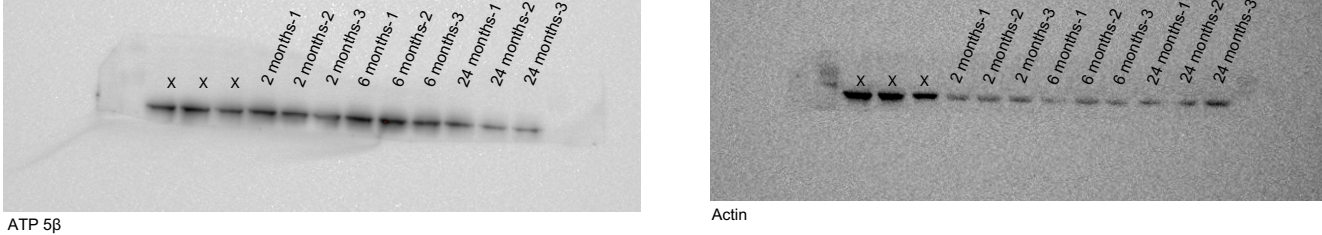

Supplement: S1 Raw Images — (PDF) [file pbio.3002753.s013.pdf]
